# Developing EFL Teachers' Technological Pedagogical Knowledge Through Practices in Virtual Platform

**DOI:** 10.3389/fpsyg.2022.916060

**Published:** 2022-05-31

**Authors:** Yu Zhang

**Affiliations:** School of Foreign Languages, Zhejiang University City College, Hangzhou, China

**Keywords:** EFL teachers, technological pedagogical knowledge, virtual platform, information and communication technology, learning communities

## Abstract

Currently, advancements in information and communication technology (ICT) and an increased interest in using the Internet for educational purposes have led educators to work in new virtual settings. However, using technology in teaching requires the understanding and information of English educators. Technological Pedagogical Content Knowledge (TPACK) is an educator's knowledge concept regarding integrating technology into education. Also, how educators integrate technology effectively into their classes is a significant issue, as learning environments are influenced by rapid advances in instructional technology. Consistent with this issue, it is critical for teacher development to integrate ICT into educational tasks in the design, application, and assessment of practices in a virtual setting. To this end, the objective of the present review is to consider the role of practice in the virtual platform in developing EFL teachers' TPACK. Ultimately, some implications are presented for the EFL educational stakeholders.

## Introduction

The advent of globalization has increased the significance of English as a universal language. It is thus essential to design EFL classes in ways that allow students to develop the necessary skills to communicate with humans who speak a variety of languages (Hsu, 2016). One of the major issues for instructional reform and innovation over the last several decades has been the integration of technology into teaching (Derakhshan et al., 2021), which is possible only by supporting learners in obtaining adequate English proficiency through ICT. Technology has continually brought about changes and difficulties in twenty-first-century English education and communication to the extent that it allows graduate students to become qualified and competent experts (Phumpho and Nomnian, 2019; Suebwongsuwan and Nomnian, 2020). One method wherein scholars attempt to comprehend how educators may better integrate technology into their classes is to concentrate on the types of information that educators need to employ effectively. Technology may enhance learners' learning, aid learners and parents by stimulating their interests, increase the school's engagement and relevance for the students, offer identical possibilities to deprived learners, and assist teachers in their professional development (Lee and Lee, 2014). However, the way educators may integrate technology into their classroom has become a crucial subject recently, as studies carried out in various settings and situations about academic technology have concluded that technology incorporation into academia is an intricate cycle (Mishra and Koehler, 2006). According to the findings of some studies in the literature (Canbazoglu Bilici et al., 2016; Janssen et al., 2019), a proper mixture of material and education with technology allows educators to efficaciously integrate technology into their teaching. Indeed, the increased accessibility of technology in schools alone does not routinely ensure better usage and helpfulness. Educators should be instructed to use it in the best way possible to aid learners' learning. Such particular information to use technology optimally to help learners' achievement in the subject is called (TPACK) (Mishra and Koehler, 2006). Indeed, TPACK is among the numerous theoretical systems expanded on in academic technology studies that arise as a system integrating the basic dynamics of teaching tasks utilizing technology (Archambault and Barnett, 2010). The significance of the association between certain TPACK elements and technology incorporation was also documented, mainly between pedagogical knowledge (PK) and ICT incorporation (Aslan and Zhu, 2017), TPACK and the intent to utilize Web 2.0 (Teo et al., 2018), and PK, technological knowledge (TK), and technology incorporation (Taimalu and Luik, 2019).

Concerning the use of technology in classrooms, Mishra and Koehler (2006) suggested a framework for teachers called TPACK. This framework is derived from the pedagogical content knowledge (PCK) structure, which refers to a theoretical structure that educators need to realize to educate using technology (Wu, 2015). Moreover, PCK mainly means that learning to teach a specific topic requires comprehension of the materials as well as designing proper teaching strategies and competencies for students (Koehler et al., 2014). TPACK is proposed as successful teaching through technology. In the existing literature, Angeli and Valanides (2005) have characterized TPACK as an important knowledge base that preservice educators must develop. As stated by Koehler et al. (2007), creating and employing effective teaching demands an understanding of how technology is associated with pedagogy and materials. Technology will stay as a peripheral ancillary to an educator's teaching unless he or she regards it as an important component of the education cycle. Actual incorporation can only be comprehended as the crossing of numerous kinds of educator knowledge (Pierson, 2001; Mahmoodi et al., 2021). Mishra and Koehler's (2006) system for TPACK is one of the most accepted and vastly studied theoretical systems for technology incorporation in the classes. They added the element of technological knowledge and proved how numerous types of teacher knowledge can be derived from the incorporation of technological, pedagogical, and content knowledge. Teacher trainers are starting to utilize the TPACK system as a lens for investigating the methods by which educators incorporate technology into the class arrangement (Hofer and Harris, 2010). Some have suggested teaching technology to educator participants in the setting of a design issue. By doing so, the candidates are at the same time introduced to the intricacies and interconnected nature of these forms of knowledge (Koehler et al., 2007; Koehler and and Mishra, 2009).

The model of TPACK is an optimal theoretical structure for extensively describing how teachers can use technology to facilitate education (Dong et al., 2015), and it refers to the information explaining educators' knowledge of the usage of technology for education (Niess, 2008). One of the most effective ways to expand educators' TPACK is to dynamically involve them in the layout of a technology-improved learning setting (Koehler et al., 2014). Undeniably, technology is considered an information system with its prejudices and affordances, making certain technologies more practical in certain settings than others (Kozikoglu and Babacan, 2019). The TPACK structure is still developing and can be turned into a learning model developed inside the teaching world, specifically in universities. Internet-based modeling has become an option and a choice for implementing this structure (Simarmata et al., 2018). E-learning creates a virtual worlds wherein students can participate in dynamic and innovative learning with others through simulating, role-playing, remote management of truly universal instruments, Internet-based learning courses, or collaboration with different academic providers (Buckingham, 2008). The activity of lesson planning based in a virtual platform is an excellent chance for educators to be dynamic technology designers and link technology with education (Lee and Lee, 2014). In a virtual setting, educators are supposed to go beyond the conventional method of providing information to alter and affect incentive, cognitive, and efficient procedures (Ferney and Castañeda, 2012). Educators designed those courses as virtual apps in an attempt to integrate technological instruments, namely, Web 2.0 (Abdelhafez, 2018).

Consistent with the review of literature, a large body of research concluded that although educators employed diverse technological tools dynamically in their after-school lives, they did not use them adequately in their classroom or make the time for such targets (Erdemir et al., 2009; Anderson and and Dron, 2017). Several educators have resisted integrating technology into their classes because of the failure of professional development programs to provide them with the needed competencies and knowledge to integrate technology into their classes efficiently. Thus, in preservice professional development classes, TPACK improvement appears crucial when considering the fact that TPACK is an essential structure that can enhance educators' education in an Internet-based class. All language educators have to become meta-cognitively conscious of their knowledge of technological educational knowledge, TPACK, and technological material knowledge (Hughes and Scharber, 2008). It is believed that preservice educators generally use technology for easy tasks, namely, sending emails and giving presentations, instead of more educational objectives (Ozer, 2018). Therefore, technological integration into educator training programs needs to be put into consideration. Integrating a learning control system into educational activities needs both specific technical information and new educational information (Ouadoud et al., 2018). Consequently, designing and developing educators' TPACK is important before integrating specific technology for learning. Regarding the critical effect of educators in a virtual environment, providing TPACK is highly significant (Kim and Hannafin, 2011). A lack of such an information base, technology choice, and integration into educational/learning activities may be conducted voluntarily or improperly, hence limiting the effectiveness of virtual apps (Guichon and Hauck, 2011). Thus, the purpose of this review is to investigate the development of such knowledge among teachers through practices in a virtual setting.

## Review of Literature

### Technological Pedagogical Content Knowledge

The source of TPACK can be traced back to Shulman's concept of pedagogical content knowledge (PCK) (Mishra and Koehler, 2006), which is primarily concerned with the growth of the most appropriate teaching performances and elements. During a debate on PCK, Shulman (1986) addressed the opposing notion of teaching material with overall pedagogical methods vs. teaching with material-specific pedagogy. This debate was founded on the historical growth of education, which contended that materials and pedagogy were involved as a single body of comprehension. With reference to these significant PCK assumptions, TPACK was presented as a system explaining the elements of successful technology integration into teaching activities (Mishra and Koehler, 2006; Schmidt et al., 2009). The suitable method for technology incorporation into this system required educators to have a conceptualization created by taking into consideration the relationships among the elements: technology, materials, and pedagogy (Angeli and Valanides, 2009). TPACK is regarded as a good way of teaching with technology that is used in a positive way to clarify the content, raise awareness of what makes a perception hard or easy to acquire, and demonstrate how technology can overcome some problematic issues during the learning process (Koh et al., 2017).

The concept of TPACK came into existence as an effective method for the conceptualization of studies and is used in classes that are equipped with technology (Koehler et al., 2014). The definition provided for TPACK includes the effective usage of technology in an educational strategy as an educational instrument, which involves using strategies that include technology to improve education and learning materials. During the educational process, educators must determine the most appropriate technological instrument to teach a subject (Mishra and Koehler, 2006). Scholars often use this notion to define the expertise needed for learning in this virtual era (Chai et al., 2013). To define the dynamic relationship between material knowledge, academic knowledge, and technological knowledge, Mishra and Koehler (2006) established the TPACK concept. In addition, such an agenda can be considered as the rising form that appropriately transcends these three elements, and this agenda shapes the basis of the education career these days. Three central mechanisms, which are considered compulsory for the dynamic use of technology in learning and teaching settings, are highlighted. These components are “Technology Knowledge,” “Pedagogical Knowledge,” and “Content Knowledge” (Mishra and Koehler, 2006; Richards, 2011). Besides new technologies such as PCs, the Internet, and videos, technology knowledge also includes widely used boards, textbooks, and projectors (Schmidt et al., 2009).

According to Schmidt et al. (2009), Content Knowledge (CK) and Pedagogical Knowledge (PK) consist of educators' understanding of education and learning procedures in the class. The term “content knowledge information” refers to what EFL educators must understand about the subject they teach, in this case, language education, and it accumulates information that cannot be shared by educators in different fields (Richards, 2011). Pedagogical knowledge refers to educators' knowledge of education and learning procedures, activities, or approaches. It encompasses academic goals, values, and purposes. This general category of information consists of overall classroom control competencies, and knowledge regarding the strategies or approaches employed in the classrooms to design lessons and evaluate students (Koehler and and Mishra, 2009). In addition, they described teaching material information as educational content knowledge and recognized educational content and information as distinct elements of information in education. Pedagogical knowledge refers to educational approaches and procedures; it consists of knowledge about classroom management, evaluation, lesson planning, and learners' learning. With this knowledge, the preservice educators choose the approach most appropriate for their subject. Contrary to the lesson, there is no explanation concerning educational approaches in the syllabus. This is because the syllabus consists of instructions for educational content for one semester, whereas the lesson plan is an instruction for educational content for one session (Schmidt et al., 2009).

Technological Pedagogical Knowledge (TPK) calls for an understanding of the general academic strategies used in integrating technology (Margerum-Leys and Marx, 2002). It requires an understanding of how education and learning will be altered by the use of specific technologies. It entails integrating technological instruments and facilities with appropriate academic designs and strategies by recognizing their strong points and constraints. The most famous PC software is designed for purposes other than academic ones (Koehler and and Mishra, 2009). That is, they are generated for trading, entertaining, communicating, and engaging in social-interaction goals. Therefore, educators need to go beyond the general usage of such technology and integrate it into education. Technological knowledge is the knowledge of standard technology and higher-level advanced technology, encompassing the competencies needed to perform particular technologies (Mishra and Koehler, 2006). In addition, all the preservice educators developed their educational content through Comic Lite, PowerPoint, Camtasia, Filmora, Canva, net, and Audacity. This was shown by the steps of “examine” and “discover,” where they taught their learners without traditional technology and instead used more advanced technology. Additionally, all preservice educators created their own educational clips, with the professors performing as their advisers in creating the clips and defining the procedure for creating clips depending on the innovation of each preservice educator.

Technological Content Knowledge (TCK) enables educators to envisage examples in which technology can be effectively integrated into their education (Margerum-Leys and Marx, 2002). For instance, important improvements could be recognized in the fields of physics and math through computer simulations (Koehler and and Mishra, 2009). This kind of knowledge indicates that technology and materials have an effect on and assist each other. Thus, educators need to be aware of both their material fields and the use of specific technologies that enhance learners' learning. This knowledge entails educators comprehending that using particular technologies that can alter the manner in which they comprehend notions in a certain material (Schmidt et al., 2009).

Pedagogical Content Knowledge (PCK) entails knowledge about which teaching methods are appropriate for the material, as well as an understanding of how various aspects of the material can be organized for improved education (Mishra and Koehler, 2006). It consists of information about linking various beliefs depending on the material, activating students' prior knowledge, taking other education tactics, and learning students' learning tactics. It is a mixture of educational material and knowledge of the corporation, representation, and compatibility of specific subjects and problems with the various learners' interests and skills, which are transferred through education. Therefore, educational content and knowledge is an easy approach to assessing the comprehension of expert material among educators (Aisyah and Munir, 2021). PCK refers to the process of imparting knowledge used in a certain field (Harris et al., 2009; Koh and Divaharan, 2013). It is required to convert the material into education, such as by presenting a topic in distinctive methods or by adapting educational content to the needs of learners and optional thoughts. This strengthens the relationships between curriculum, evaluation, and education. Simultaneously, it seeks to develop learning material in the same way as that in English teaching; educators with a TPACK viewpoint are required to comprehend the right pedagogy notion for incorporating technology into education and learning tasks. In this way, learners are engaged and encouraged to dig deeper into the English learning material. Using proper TPACK, educators can motivate learners to investigate English learning material. The model indicates that CK, which combines technology and educational capabilities, is vital for facilitating efficient and innovative classroom education (Mishra and Koehler, 2006).

The structure of TPACK could offer notional serve as a model agenda for designing courses that can integrate all areas of education of all the fields of educator knowledge: material (subject), education (educating and learners' learning), and technology (Mishra and Koehler, 2006; Lyublinskaya and Tournaki, 2011). TPACK is defined as the knowledge that educators require to educate students using education through technology in their particular material fields and grade levels (Niess, 2008). For using the TPACK framework in technology integration, educational institutes ought to have access to technology sources, an appropriate curriculum, and genuine experiences regarding using technology to integrate tasks and theories into particular educational programs for educator training. They were willing to use TPACK during the educational learning procedure despite the fact that they acknowledged some drawbacks associated with the usage of PCK, TCK, and TPK, one of which is the problem of creating an academic clip. Additionally, they required several advancements that were pertinent to the topic and the clip. Debreli (2016) believed that preservice educators gain new experiences to become “actual educators” and also confront problems during education in the class. The professor in this study provided feedback according to their educational competencies in the future. Therefore, to use TPACK, educators need to be equipped with gadgets, which include a laptop, a projector, and an energy network, due to the fact that TPACK includes technology to improve the framework of TPACK. Moreover, Shadiev and Yang (2020) recommended incorporating technology into the education teaching and learning process, where educators could regulate their educational tasks, as well as their educational tactics, to effectively employ the present sources in the best way possible (Tondeur et al., 2012; Basirat and Taghizadeh, 2021).

### Virtual Professional Learning Communities

The community notion is important for comprehending the ways people learn and how professional development can occur virtually. In line with the theory of social learning proposed by Wenger et al. (2002), there is a growing need for new professional development possibilities and venues as each of us becomes a more connected member of the global community. These days, it is rare to find a professional development project of any size or duration that does not incorporate at least one or more general Internet technologies to nurture and boost conversation and/or knowledge sharing (Schlager and Fusco, 2003). Virtual professional development societies grounded in a community framework can transform educators' professional development by assisting educators in learning about how to increase appropriate academic and educational evolutions, the embedded transformation of education with merging technology. Robust virtual groups enable remote subjects to feel a sense of connection with the whole group (Wenger et al., 2002). Internet-based learning is referred to as e-learning by using online technology as a learning method. The benefits of e-learning consist of (1) saving time for the process of educating and learning; (2) lowering trip charges; (3) saving general teaching prices (facilities, infrastructure, textbooks); (4) achieving the entire geographical region; (5) and teaching self-dependent learning (Bora and Ahmed, 2013). The virtual setting laid the groundwork for educators to collaborate on improving their efficiency, sharing learner information, solving problems, implementing educational innovations, and repeating the cycle with similar material mates. Presenting a virtual setting that makes use of an internet-based meeting environment lays the groundwork for educators to collaborate and consequently resolve issues of educator isolation from the school environment (McConnell et al., 2013). The virtual setting enables educators in these disciplines to overcome the geographic constraints that lead to educator isolation (McConnell et al., 2013).

Effective teaching with technology is a multi-faceted cycle that demands (1) an understanding of the portrayal and articulation of notions utilizing technologies; (2) pedagogical strategies that use technologies in positive methods to instruct material; (3) knowledge of what makes notions hard or simple to study and how technology can address these problems; (4) knowledge of learners' past knowledge and hypotheses of epistemology; and (5) an understanding of how technologies can be used to develop on present knowledge and to create novel or enhance past epistemologies (Koehler et al., 2007).

## Conclusion, Implications, and Suggestions for Further Research

The present review sets out to illustrate the development of TPACK through practices in virtual settings, specifically how it is used to specify teachers' qualifications and skills in online learning, which is currently essential in language learning. The review of the related study will be remarkably important because attaining high-quality education in Internet-based courses requires comprehending the ways various fields of knowledge concerning material, education, and technology meaningfully interact and what notions and perspectives successful educators possess for subsequent training in their professional development programs. Being capable of training with technology needs knowledge of the way technology, pedagogy, and material have interacted to aid learners' learning. Put simply, it includes the competence and information to be able to take advantage of a virtual instrument or app with all its capabilities, constraints, and potential to help learners' learn a subject or material provided. TPACK may support teachers to comprehend the possible assistance of new technologies in education, and using the TPACK structure is vital in the field of English language education because there are many reasons for this; in EFL situations, meeting the goal language outdoors may be limited. Virtual technology presents valid English. As stated by Liu et al. (2014), for EFL educators, the manner of using the technological instrument is a section of their expert information about education.

Technology can motivate educators to implement learner-oriented learning by using appropriate material, education, and technology. In agreement with the findings reported in the literature review, the advantages of this kind of technology educational course for educators can be observed more in terms of viewpoint than specific competencies or information. Consequently, a properly designed educational course ought to highlight subjects' development of assurance and tendency to apply and make use of technology in language classes. This could be accomplished by developing clearly articulated course goals and rationales for technology integration, as well as emphasizing the numerous benefits of integration. It is also vital to debate social and circumstantial constraints and problems that limit technology in the classroom and feasible solutions so that subjects have a more realistic view of what they can and cannot achieve in special environments. Additionally, it is crucial to take into account educators' backgrounds and rely on their educational and technological experience; the course may also depend more on the subjects' technological competencies or educational knowledge, even if they are both identical. Different circumstantial elements, including class equipment and academic guidelines, need to be carefully considered when designing a circumstantially suitable technology educational course that promotes local language education.

Investigating the book content accessible online can improve the subjects' comprehension of ICT merging, hence aiding the TPACK improvement. Accessing digital settings through specific activities and tasks appears to improve preservice technology educators' viewpoints, skills, and confidence about the integration of ICT in education and learning, which, in turn, aids TPACK development. Additional insights into Internet-based courses are required to direct the design of future educator-taught courses and educators who are more ready to teach in virtual settings. Through teaching in virtual environments, educators are assisted in finding out that successful technology integration is feasible, which results in educators' implementation of technology integration. It shows that this form of activity as an expert improvement program can significantly develop EFL educators' TPACK literacy. TPACK educational programs in virtual apps aid educators in broadening their belief in the practical competencies required for technology integration. The most significant aspect of this research is that teaching in a virtual environment is a successful educational program, especially for EFL educators, because it enables them to develop their TPACK competencies, apply those competencies in education, and be aware of TPACK merging elements. The subject is critical because EFL students in the twenty-first century cannot avoid using technology in their classes. Consequently, educators need to learn how to practically apply their technical knowledge because the final examination of knowledge relies on one's capability to convert information into education. Also, it seems vital for teacher trainers to inform their teachers about the role of TPACK in designing actual teaching methods with technology to fulfill the desires of twenty-first-century learning abilities. More studies, especially empirical studies, should be conducted to consider observations or interviews to consider the role of such a platform in developing TPACK and getting a better representation of EFL teachers' present technological knowledge. Internet-based educators state that they need extra help in terms of education and pedagogy (Morris and Finnegan, 2008; Oomen-Early and Murphy, 2009). It is said that TPACK features provide prospective educators with a knowledge framework and associated competencies for becoming aware of technology usage to adapt internet learning settings for increased effectiveness and genuine—rather than rote—learning regarding the usage of technology for overall goals (Mishra and Koehler, 2006).

Educator trainers confronted with the task of redesigning their programs to include more tasks and activities are supposed to pay attention to educating new educators on how to integrate technology to enhance and distinguish learners' learning. Courses that integrate the education of educators' knowledge in all the fields are essential: knowledge of content, education, and technology. Learning technical competencies alone is not enough—learning how to integrate technologies into teaching is equally essential. Further studies are recommended to determine the extent to which educator preparedness programs equip new educators to use technology effectively to increase the learning of all learners. Therefore, with continuing critical preparation of new educational methods in teaching, TPACK can be an innovative method for evaluating educational knowledge and its power to affect teacher educators and their ability to enhance their profession. Preparing future educators requires transitioning from conventional methods to advanced ones, such as integrating technology into content classes. Conducting workshops and educational seminars on the TPACK framework of knowledge and competencies, as well as examining the effects of this education on educators' knowledge and activities, is motivated more by long-term effects and the possibility of a change in the quality of Internet-based education. To elicit strong perspectives from educators and educator trainers regarding vital knowledge and competencies for internet-based education, we suggest several data collection sources, such as group discussions, conducting interviews, keeping personal journals, and conducting self-report investigations.

## Author Contributions

The author confirms being the sole contributor of this work and has approved it for publication.

## Funding

This work was supported by The 2nd Batch of Teaching Reform Research Project of Thirteenth Five-Year Plan of Higher Education in Zhejiang Province, 2019.

## Conflict of Interest

The author declares that the research was conducted in the absence of any commercial or financial relationships that could be construed as a potential conflict of interest.

## Publisher's Note

All claims expressed in this article are solely those of the authors and do not necessarily represent those of their affiliated organizations, or those of the publisher, the editors and the reviewers. Any product that may be evaluated in this article, or claim that may be made by its manufacturer, is not guaranteed or endorsed by the publisher.
